# Cell signaling and cytomegalovirus reactivation: what do Src family kinases have to do with it?

**DOI:** 10.1042/BST20191110

**Published:** 2020-04-20

**Authors:** Matthew B. Reeves

**Affiliations:** Institute of Immunity and Transplantation, University College London, Royal Free Campus, London NW3 2PF, U.K.

**Keywords:** cell signaling, chromatin, cytomegalovirus, hematopoiesis, latency, Src family kinases

## Abstract

Primary infection with human cytomegalovirus (HCMV) is usually asymptomatic and leads to the establishment of lifelong latent infection. A major site of latency are the CD34+ hematopoietic progenitor cells. Importantly, normal cellular differentiation of CD34+ cells to a macrophage or dendritic cell phenotype is concomitant with viral reactivation. Molecular studies of HCMV latency have shown that the latent viral genome is associated with histone proteins and that specific post-translational modifications of these histones correlates with the transcriptional activity of the genome arguing that expression of key viral genes that dictate latency and reactivation are subject to the rules of the histone code hypothesis postulated for the regulation of eukaryotic gene expression. Finally, many studies now point to a key role for multiple signaling pathways to provide the cue for HCMV reactivation. The challenge now is to understand the complex interplay between cell identity, transcriptional regulation and cell signaling that occurs to promote reactivation and, additionally, how HCMV may further manipulate these events to support reactivation. Understanding how HCMV utilizes these pathways to drive HCMV reactivation will provide new insight into the mechanisms that govern viral and host gene expression and, potentially, illuminate new, host-directed, therapeutic opportunities to support our attempts to control this important medical pathogen of immune-compromised individuals.

## Introduction

Human cytomegalovirus (HCMV), a large DNA virus, is a member of the herpesvirus family. Like all herpes viruses, HCMV has the capacity to establish lifelong latent infections of the host. The simplest definition of viral latency is defined as the persistence of the viral genome in the cell with an absence of lytic replication and virion production but the genome retains the capacity to reactivate and re-enter the lytic lifecycle when specific conditions are met.

Reactivation poses a unique set of problems for any virus capable of establishing latency. Upon primary infection, the virion delivers many pre-formed proteins into the cell that play key roles during the initial stages of infection: These incoming proteins transactivate viral gene expression and also disable cell-autonomous immune functions that otherwise would limit infection [1,2]. Thus the virion comes ‘pre-loaded’ with the functions required to initiate infection which, in the case of HCMV, requires the activation of the major immediate early promoter (MIEP) and production of the associated MIE proteins essential for driving lytic infection. Furthermore, it is likely that signaling events associated with pathogen binding and entry contribute to generating a cellular environment conducive for the initiation of lytic gene expression [3]. In contrast, the re-animation of viral lytic gene expression from a latent viral genome likely occurs in an absence of at least a majority of those events and functions associated with the incoming virions. Furthermore, the latent genome is highly compacted due to an association with heterochromatin providing a further barrier to reactivation of gene expression (see later sections). What is becoming increasingly clear is that the virus relies on host cues for reactivation which, via the expression of a subset of latent gene products, HCMV further manipulates to promote efficient viral reactivation.

A wealth of studies from multiple groups have led to a model of HCMV latency and reactivation that is dependent on many important observations:

(i) A major site of HCMV latency are the hematopoietic progenitor cells resident in the bone marrow which give rise to cells that populate the blood [4–8].
(ii) The carriage of latent viral genomes is predominantly in cells of the monocyte/myeloid lineage which culminates in viral reactivation in the macrophage and dendritic cell (DC) lineages [7,9–16].
(iii) In natural latency, multiple copies of the HCMV latent genome persist as circularized, episomal genetic units in the nucleus of the host cell [17]. Furthermore, these viral genomes are associated with host chromatin [10].
(iv) The association with chromatin is dynamic. Specifically, it has been demonstrated that the MIEP is associated with transcriptionally repressive chromatin in latently infected cells but, in cells that support reactivation, the histones at the MIEP are acetylated consistent with the transcriptional activity observed [10,18–20].
(v) Inflammation (and the associated signaling activity) promotes HCMV reactivation in multiple models of HCMV latency [7,11,21–24].

Accordingly, it is hypothesized that viral genomes are regulated by chromatin structure and, specifically, host cell changes associated with cellular differentiation of myeloid progenitor cells into DCs or macrophages are likely responsible for promoting the post-translational modification of histones required for gene expression to occur. Furthermore, these changes are driven by the activity of host cell signaling pathways. Essentially, the viral genome has become subject to the same mechanisms governing the differential regulation of eukaryotic gene expression that also occurs in a cell type and ligand-specific manner.

In this short review, I will briefly introduce the evidence that supports the importance of host cell signaling which, when acting in concert with host cell differentiation, promotes viral lytic gene expression and reactivation. Although the focus is on the role of Src family kinase (SFK) signaling in interleukin-6 (IL-6) induced reactivation, I will also discuss the importance of viral gene products in manipulating multiple cell signaling pathways for the establishment of latency and, subsequently, demonstrate the increasing complexity that underpins the molecular mechanisms controlling HCMV reactivation.

## Latent HCMV genomes are regulated by host chromatin

The work of our laboratory has focused on delineating host functions important for HCMV reactivation. Our studies are predicated on the assumption that the latent HCMV genomes are under the same constraints of the host genetic material and, therefore, likely subject to the same host-mediated mechanisms of gene regulation. Indeed, the demonstration that the latent viral genome is associated with chromatin in naturally infected individuals provides a clear mechanism for the control of HCMV latency and reactivation [10]. A reductionist view of latency and reactivation is that it is hallmarked by differential regulation of major lytic gene expression (i.e. MIE gene expression). Thus, given that the viral genome is associated with cellular chromatin, the histone code hypothesis postulated for the control of cellular gene expression by post-translational modification of histone proteins [25] becomes applicable to the regulation of viral gene expression also.

**Figure 1.**
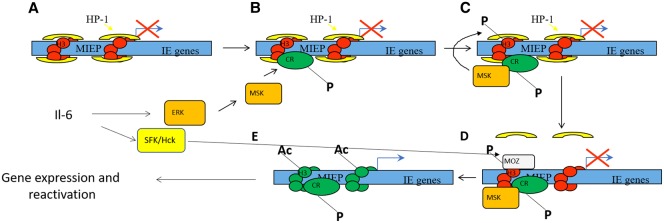
Model for IL-6 induced reactivation in dendritic cells. The MIEP of HCMV is associated with heterochromatin in latently infected CD34+ cells (**A**). Concomitant differentiation to a dendritic cell phenotype and activation of inflammatory IL-6 signaling initiates ERK–MAPK and SFK signaling. The activation of ERK promotes activation of mitogen and stress-activated kinases 1 and 2 (MSKs) which recognize the CREB transcription factor bound to the MIEP (**B**). Recruitment of MSK via CREB to the MIEP promotes dual phosphorylation of CREB and also histone H3 at serine 10 (**C**). Phosphorylation of serine 10 de-stabilizes the binding of HP-1 to methylated lysine 9 residues promoting its dissociation from the MIEP (**D**). This activation of SFK promotes the recruitment of MOZ histone acetyltransferase activity to the MIEP (**D**) which drives histone acetylation facilitating the robust induction of MIE gene expression (**E**) — the first event in full viral reactivation.

In support of this concept is the observation that the chromatin signature associated with the viral genome at the MIEP correlates with the pattern of gene expression observed (Figure 1A,E). Specifically, in latency where MIE gene expression is repressed the MIEP is associated with histone H3 methylated on lysine residues 9 and 27 and the transcriptional repressor heterochromatin protein-1 (HP-1) [10,18,20,26]. In contrast, in reactivating cells where MIE expression is detected the MIEP is associated with acetylated H3 and H4 histones [10,20,26]. However, whilst instructive this does not explain the mechanism of how these changes in chromatin phenotype occur — just that they do occur. Since the same mechanistic question of how chromatin is modified applies to the regulation of host gene expression by chromatin parallels can be drawn.

Thus central to understanding the mechanisms governing HCMV reactivation was to first identify the triggers of HCMV reactivation in differentiated myeloid cells.

## Inflammation-induced cell signaling promotes HCMV reactivation

The first clues that inflammation-associated cell signaling was likely important for viral reactivation came from many studies performed over 20 years ago. The demonstration that both CD34+ progenitors, granulocyte-macrophage progenitors and CD14+ monocytes were sites of HCMV latency [4,6,7,13,14,27] was followed up with the key observations that cellular differentiation of these cells was a driver of viral reactivation [7,10–12]. Importantly, reactivation appeared to be more efficient in the presence of inflammatory cytokine environments. These observations were entirely consistent with the architecture of the MIEP which contains binding sites for many transcription factor binding sites (e.g. NF-κB, CREB, AP-1, Stat) important for potentiating inflammatory responses [28].

To identify potential host mechanisms important for HCMV reactivation we focused on inflammation-associated signaling in DCs — a cell type that supports HCMV reactivation *in vitro* and *ex vivo* [10,16,21]. The identification of IL-6 as an important mediator of reactivation allowed us, via multiple studies, to demonstrate that IL-6 promoted HCMV reactivation via ERK–MAPK activity [21,29]. In turn, ERK–MAPK activity promoted the activation of mitogen and stress-activated kinases (MSKs). The importance of MSK activity in HCMV reactivation became apparent due to a capacity to phosphorylate both the transcription factor CREB and also histone H3 at serine 10 [29,30]. The observed histone phosphorylation at the MIEP was hypothesized to represent a crucial intermediary event that underpinned the remodeling of the chromatin at the MIEP observed in cells supporting HCMV reactivation since histone serine 10 phosphorylation de-stabilizes the interaction of HP-1 with methylated histones [31]. Additionally, it became clear the recruitment of MSKs to the MIEP was dependent on the CREB transcription factor binding sites in the MIEP [29]. Thus MSK activity in a DC was only pro HCMV lytic gene expression during the reactivation phase if CREB was bound to the target promoter (Figure 1B,C).

## Multiple cell signaling pathways have been implicated in HCMV reactivation

Clinical studies quite clearly demonstrate the importance of HCMV reactivation *in vivo*. Indeed, in bone marrow transplant patients over 80% of seropositive transplant recipients will have evidence of HCMV viraemia which, if left untreated, can cause severe morbidity [32]. The frequency of clinical HCMV reactivation also argues that this virus has a proclivity to reactivate *in vivo* given the right signal or, indeed, the right signals. Historic studies demonstrated that TNF-α was pro-reactivation — which was hypothesized to promote the recruitment of the NF-κB transcription factor to the MIEP [22]. Indeed, transcription from the MIEP has been demonstrated to occur in response to multiple inflammatory stimuli including IL-6, TNF-α, IL-1β and prostaglandin E2 again linking HCMV reactivation with inflammation-associated signaling [33,34].

Additionally, Pi3K signaling has been linked to the regulation of HCMV reactivation through the activity of a virally encoded latent gene product, UL138 [35]. This protein has been reported to have an antagonistic relationship with another viral protein, UL135 [36]. During latency, UL138 regulates epidermal growth factor receptor (EGFR) localization to ensure Pi3K signaling is active in the cell which is hypothesized to help maintain latency via the Egr-1 transcription factor [37] — an event also targeted by a virally encoded miRNA [38]. Consistent with this is the observation that Pi3K inhibitors trigger HCMV reactivation *in vitro* suggesting that this pathway actively supports latency [37]. Interestingly, work from the Trono laboratory demonstrated that chloroquine also drove HCMV reactivation by modulating the activity of the transcriptional repressor, TRIM28/KAP1 [39]. This was linked to mTORC1 activation but it is of note that chloroquine also inhibits Pi3K activity suggesting that the effects of chloroquine may be multi-faceted. Sustained Pi3K activity has also been demonstrated to be important for the survival of latently infected cells [40]. Intriguingly, caspase activity (a major driver of apoptotic cell death) is also associated with myeloid cell differentiation [41] and, potentially, the activation of HCMV gene expression [42]. In the herpes simplex virus model of latency and reactivation in neurons loss of Pi3K signaling (via withdrawal of nerve growth factor or pharmacological inhibition) can drive reactivation [43]. Both these events can trigger cell death pathways also and thus, potentially, loss of control of cell death pathways may be contributing to the latency/reactivation phenotype in herpes viruses.

For the purpose of this review, however, is the more general implication that the study of any specific cell signaling pathway often has to be considered within the framework of the signalsome in the host cell. Thus understanding the context in which a signaling pathway is being activated becomes crucial. Signaling does not occur in isolation whereby it is highly likely that more than one pathway is going to be important for the outcomes you are analyzing — particularly given the clear cross-talk that exists within signaling pathways.

## Why does IL-6 mediated activation of ERK promote HCMV reactivation in DCs?

In our initial studies of HCMV reactivation, we remained puzzled by the observation that ERK–MAPK signaling promoted reactivation in a cell type-dependent manner [21,44]. ERK–MAPK represents a ubiquitous pathway which can be activated in progenitor cells as well as DCs yet reactivation was not triggered in the progenitor cells incubated with IL-6. Put more broadly, the question being addressed was how are cell type-specific signaling and gene expression responses imparted?

To address this, we tested whether ERK–MAPK alone was sufficient to induce reactivation. Ectopic activation with a constitutively active version of MEK (the upstream kinase of ERK) failed to robustly promote reactivation supporting the concept that the cellular context of the ERK–MAPK signaling was important [45]. Here our parallel work in monocyte-derived Langerhans-like cells (LCs) proved instructive [44]. These cells failed to support robust IL-6 induced reactivation despite a clear capacity to respond to IL-6 and, indeed, trigger substantial ERK activation. Hypothesizing that a second IL-6 induced signal was critical we used phospho-proteomics to compare monocyte-derived DCs with LCs stimulated with IL-6. Using this approach we observed a discrete set of differences including changes in Src family kinase protein phosphorylation and, intriguingly, mTOR [45].

## Src family kinases (SFKs)

The SFKs comprise nine members: Src, Yes, Fgr and Fyn (subfamily A), Hck, Lck, Blk and Lyn (subfamily B) and Yrk [46]. SFKs are non-receptor tyrosine kinases that have critical roles in normal cell homeostasis. Notably, work on the transforming potential of Rous sarcoma virus led to the identification and classification of Src as the first proto-oncogene [47]. Subsequent studies of other SFKs have also implicated them in cancer through the acquisition of mutations that promote dysregulation of host cell signaling although not necessarily leading to their classification as oncogenes *per se*.

The phospho-proteomics performed in DCs and LCs [45] revealed two SFKs, Src and Hck, demonstrated increased phosphorylation in DCs that support reactivation of HCMV. Src is ubiquitously expressed whereas Hck is largely restricted to cells of the hematopoietic lineage [48,49]. The elevated expression of Hck in DCs suggested that increased availability of this protein may underpin a cell type-specific role in HCMV reactivation and thus Hck was investigated further [45].

Hck exists in two isoforms generated from a single transcript — a lysosome-associated form and plasma membrane-associated form are evident in cells [50]. Inactive Hck is maintained in the cell by selective phosphorylation events that promote inhibitory intra-molecular structure. Key to this is the inhibitory activity of the CD45 phosphatase — a protein ubiquitous to hematopoietic cells — and also C-terminal Src kinase (CSK). Activation of Hck requires de-stabilization of the intra-molecular structure and can be initiated by engagement of LPS, IL-2, IL-6 and GM-CSF with their cognate receptors which, in turn, activate the SFKs. The key to the activity of Hck is a capacity to interact with multiple cell surface receptors to promote downstream signaling events [50]. In the context of our studies, we predict Hck interacts with gp130 — the signaling component that dimerizes with the IL-6 receptor [51]. Importantly, this interaction with gp130 has been shown to be important for the potentiation of ERK–MAPK mediated signaling in myeloma cells and thus became highly pertinent to our own studies of HCMV reactivation in DCs [52].

## SFK activity promotes events post ERK to support HCMV reactivation

The implication that Hck activity potentiated ERK–MAPK signaling potentially explained the reactivation phenotype in DCs. However, this view was tempered by the MEK overexpression data which argued that simply high levels of ERK signaling was not the driving factor [45]. Indeed, in the phospho-proteomics IL-6 appeared to promote comparable levels of ERK phosphorylation in the LCs when compared with the DCs [45]. Thus we considered the role of Hck and SFK activity was not just an amplification of ERK activity alone.

We had previously demonstrated that ERK–MAPK activity promoted histone phosphorylation at the MIEP and implicated this event in reactivation [29]. In HSV, a phospho-methyl switch has been proposed whereby histone phosphorylation allows gene expression from promoters associated with histones ostensibly classed as repressive (methylated at lysine residue 9 of histone H3) [53]. In our original studies of HCMV latency and reactivation, it was clear that histone acetylation at the MIEP was correlated with reactivation [10,20] and thus we hypothesized that a host histone acetyltransferase (HAT) activity was likely important for reactivation and that histone phosphorylation was a step required for this.

Studies of the chromatin phenotype at the MIEP and, specifically, how it changed with time in response to IL-6 proved instructive when analyzed in the context of data that measured the impact of HAT inhibitors on HCMV reactivation. What became evident was that whilst ERK–MAPK promoted histone phosphorylation at the MIEP, subsequent de-methylation and histone acetylation was impacted by a second pathway which was revealed to be SFK dependent (Figure 1D). Thus whilst ERK was responsible for initiating changes to chromatin structure at the MIEP via the activity of MSKs, these events were potentiated by the recruitment of the monocytic leukaemic zinc finger (MOZ) HAT to the MIEP in an SFK dependent manner [45].

## Viral functions manipulating cell signaling

Interestingly, the identification of a role for SFK activity during HCMV reactivation became more intriguing in light of emerging data arguing that the virus itself was not a passive observer in the process of latency and reactivation. Very recently it has been shown that an HCMV gene product, UL7, binds to flt3 ligand receptor to drive myelopoiesis and HCMV reactivation [54]. An important component of flt3 ligand signaling is the activation of SFKs [55] which may suggest a more involved role for SFKs in viral reactivation above the recruitment of MOZ to the MIEP.

A second viral gene product, US28, encodes for a GPCR that can modulate cell signaling pathways during both latent and lytic infection [56,57]. Fascinatingly, US28 can either repress or activate the same pathways in a cell type-specific manner providing HCMV with molecular tool to hijack and re-direct host cell signaling [58]. Pertinent to many studies was the identification that US28 expression in cells that support viral latency down-regulated many pathways implicated in HCMV reactivation including ERK–MAPK and NF-κB [21,22]. Furthermore, US28 activity in cells that support HCMV reactivation promoted the activation of the same host cell pathways [58].

However, this impact on cell signaling was not uniform for all pathways of interest and specifically US28 did not appear to inhibit SFK signaling in the original study [58]. Indeed, US28 promoted up-regulation of SFK signaling including SFK activity in latently infected cells. One hypothesized benefit is an SFK driven up-regulation of the trans-endothelial migration of latently infected monocytes [59]. Indeed, the manipulation of SFK signaling also occurs in B cells infected with Epstein–Barr virus (EBV) where the latent membrane protein 1 and 2A (LMP1 and LMP2A) have been shown to promote SFK signaling which contributes to cell survival and the establishment and maintenance of latency suggesting parallels with HCMV and US28 [60,61]. Furthermore, in the murine model of gammaherpes virus infection, Src kinase activity has been linked with the regulation of MHV-68 reactivation [62].

In the context of HCMV latency and reactivation, the up-regulation of SFK activity appears counter-intuitive. One potential explanation is that simply the activation of a signaling pathway is not the only important event but also the nature of the stimuli and thus the ligand:receptor pathway responsible for Hck activation. As stated above, Hck activation occurs in response to many ligands and, potentially, the nature of the stimuli dictates the downstream events it triggers [50]. Activation of Hck (or any signaling pathway) does not necessarily mean that every potential target downstream will be activated. Thus the importance of IL-6 in reactivation in DCs may be that the activation of Hck via *gp130* is crucial for reactivation-specific signaling events. Indeed, the sequestration of Hck signaling by US28 in latently infected cells may result in reducing the availability of Hck for reactivation. Furthermore, the up-regulation of Hck in DCs thus could increase the availability of Hck for IL-6 signaling and thus reactivation may explain the propensity for reactivation to occur more efficiently in DCs. These hypotheses are easily testable and will provide new insight into HCMV reactivation and also the complexity associated with cell-type-specific signaling events.

## Conclusion

The co-evolution of HCMV with the host has rendered the virus an integral component of the signaling events in the latently infected cell. This is evidenced by both a clear responsiveness to host driven cues of inflammation and cellular differentiation as well as the expression of viral gene products to further manipulate the activity of key pathways in the cells. What studies of HCMV latency exemplify is the complexity associated with interpreting the role of cell signaling pathway activity in the control of HCMV and, by extension, eukaryotic gene expression.

## Perspectives

- Is complexity in the signaling pathway usage indicative of a requirement to activate multiple promoters [63] for HCMV to reactivate? Or instead, is the virus capable of responding to multiple different stimuli rendering reactivation more likely?
- How does Hck and SFK signaling dictate the recruitment of HAT activity? Is an intermediary required?
- How important is the nature of the stimulus that activates Hck/SFK activity? Specifically, is Hck pro-reactivation only via gp130 receptor engagement?
- Can inhibition of Hck-specific functions associated with HCMV reactivation be tolerated by the host cell? Targeting host functions dramatically raises the barrier to the development of drug resistance in viral infections and thus may prove useful for future strategies to control HCMV reactivation in susceptible patient populations.

## Open Access

Open access for this article was enabled by the participation of University College London in an all-inclusive *Read & Publish* pilot with Portland Press and the Biochemical Society under a transformative agreement with JISC.

## Abbreviations

CSK: C-terminal Src kinase
DC: dendritic cell
HAT: histone acetyltransferase
HCMV: human cytomegalovirus
HP-1: heterochromatin protein-1
LCs: Langerhans-like cells
MIEP: major immediate early promoter
MSKs: mitogen and stress-activated kinases
SFK: Src family kinase
SFKs: Src family kinases
